# Geographical differences in the financial impacts of different forms of tobacco licence fees on small retailers in Scotland

**DOI:** 10.1136/tc-2023-058342

**Published:** 2024-02-07

**Authors:** Roberto Valiente, Helena Tunstall, Amanda Y Kong, Luke B Wilson, Duncan Gillespie, Colin Angus, Alan Brennan, Niamh K Shortt, Jamie Pearce

**Affiliations:** 1 Centre for Research on Environment, Society and Health (CRESH), School of GeoSciences, University of Edinburgh, Edinburgh, UK; 2 SPECTRUM Consortium, UK; 3 Department of Family and Preventive Medicine, The University of Oklahoma Health Sciences Center, Oklahoma City, Oklahoma, USA; 4 TSET Health Promotion Center, Stephenson Cancer Center, The University of Oklahoma Health Sciences Center, Oklahoma City, Oklahoma, USA; 5 Sheffield Addictions Research Group, School of Medicine and Population Health, University of Sheffield, Sheffield, UK

**Keywords:** Public policy, Economics, Socioeconomic status, Environment, End game

## Abstract

**Objective:**

Retailer licencing fees are a promising avenue to regulate tobacco availability. However, they face strong opposition from retailers and the tobacco industry, who argue significant financial impacts. This study compares the impacts of different forms of tobacco licence schemes on retailers’ profits in Scotland.

**Methods:**

We calculated gross profits from tobacco sales in 179 convenience stores across Scotland using 1 099 697 electronic point-of-sale records from 16 weeks between 2019 and 2022. We estimated different fees using universal, volumetric and separate urban/rural schemes. We identified the point at which 50% of retailers would no longer make a gross profit on tobacco sales for each scheme and modelled the financial impact of 10 incremental fee levels. The financial impact was assessed based on changes in retailers’ tobacco gross profits. Differences by neighbourhood deprivation and urban/rural status were examined.

**Results:**

The gross profit from tobacco per convenience store averaged £15 859/year. Profits were 2.29 times higher in urban (vs rural) areas and 1.59 times higher in high-deprivation (vs low-deprivation) areas, attributable to higher sales volumes. Tobacco gross profit decreased proportionally with increasing fee levels. Universal and urban/rural fees had greater gross profit reductions in rural and/or less deprived areas, where profits were lower, compared with volumetric fees.

**Conclusion:**

The introduction of tobacco licence fees offers a potential opportunity for reducing the availability of tobacco retailers. The likely impact of a tobacco licence fee is sensitive to the type of licence scheme implemented, the level at which fees are set and the retailers’ location in relation to neighbourhood deprivation and rurality.

**WHAT IS ALREADY KNOWN ON THIS TOPIC**

- The WHO Framework Convention on Tobacco Control encourages the implementation of licencing systems to regulate tobacco product availability.
- Jurisdictions worldwide have considered different forms of tobacco licencing.
- The potential financial impacts of these different schemes on retailers remain unexplored.

**WHAT THIS STUDY ADDS**

- Retailers in urban and highly deprived areas had higher gross profits from tobacco due to higher sales volume.
- Licencing strategies based on flat fees (ie, universal or urban/rural) may disproportionally affect the gross profits of retailers in rural and/or low-deprivation areas, with lower tobacco sales volume.
- The magnitude of the financial impacts of schemes on retailers was significantly modulated by the fee level.

**HOW THIS STUDY MIGHT AFFECT RESEARCH, PRACTICE OR POLICY**

- Tobacco licence schemes are a potential mechanism for disincentivising the sale of tobacco products and reducing the local availability of tobacco retailers. However, the likely impact of tobacco licences is heavily affected by scheme type, fee levels and retailer location.
- Policymakers should recognise the spatial differences in the distribution of retail tobacco sales and gross profits to ensure the financial impacts of the introduction of a tobacco licence scheme benefit local population health without disproportionately impacting the business model of smaller retailers.
- Our findings underscore the importance of future tobacco retail reduction policies to provide economically viable alternatives to support retailers in diversifying their business models away from tobacco.

## Introduction

 The implementation of retail licencing systems—whereby jurisdictions require retailers to purchase a special licence to legally sell tobacco^1^—is often considered as a key component for achieving the tobacco endgame.^2^,^4^ They are crucial to monitor retailers selling tobacco, ensure trading standards, reduce illicit sales and strengthen other regulations such as sales prohibition to minors.^5^ Attaching fees to licencing systems can improve their enforcement and increase the cost of selling tobacco, making it less profitable for retailers, and in turn reduce tobacco availability and smoking prevalence as retailers choose to stop selling tobacco.^6^ However, there is a delicate trade-off between the benefits of using fee-based licence systems and their potential financial impacts on the retailers and local communities.

Fifty-three countries have some form of tobacco licencing system, frequently structured around universal, volumetric or urban/rural fee schemes.^7 8^ Universal fees refer to single flat fees imposed on retailers regardless of their characteristics or location. New Brunswick, Canada ($C100 initial fee and $C50 annual renewal),^9^ Western Australia ($A317 the first year and $A270 annual renewal)^10^ or Finland (€100 initial fee and €500 annual renewal) are examples of countries using universal fees.^11^ In contrast, a volumetric scheme levies proportional fees to retailers’ tobacco sales volume or revenues, while urban/rural schemes referred to differential flat fees for tobacco retailers in urban and rural areas. For example, both volumetric and urban/rural licencing approaches are employed in Spain’s Tobacco State Monopoly, where retailers pay a differential flat fee depending on the population size of the settlement in which they operate (€240.40 for settlements with >100 000 residents, €180.30 for settlements with 10 000–100 000 inhabitants or €120.20 for settlements with <10 000 people) as well as a volumetric fee that equates to between 1.2% and 2.1% of their revenues.^12^,^15^

The implementation of licence fees has been challenged by retailers and the tobacco industry raising concerns regarding their impacts on business profitability.^1 16^ However, no previous studies explored the extent to which different fee structures affect retailers’ profits and the local economy.^17^ Understanding such impacts is particularly crucial in rural and socioeconomically deprived communities, where small retailers play an important role in economic and social development.^18^ Additionally, it is essential to assess the effectiveness of the different fee schemes in potentially motivating retailers to divest from tobacco sales.^19^

The focus of the current study is Scotland where, since 2010, retailers willing to sell tobacco have been obliged to register as tobacco sellers for free. Recently, the Scottish Government committed to achieving a smoke-free generation by 2034 (defined as less than 5% of all adults smoking), and the introduction of a fee scheme on the current registration system is under active consideration as a policy that could help to reduce smoking rates.^20^ The present study aims to compare the potential financial impacts of different forms of fee schemes to regulate tobacco sales of retailers in Scotland.

We use transaction data from a sample of retailers to sequentially investigate three specific objectives. First, we quantify to what extent tobacco sales are important for retailers by calculating financial indicators, including annual *tobacco sales volume* and *gross profits*. Second, we estimate potential *policy scenarios* representing the implementation of different levels of fees under universal, volumetric or urban/rural schemes. Lastly, we estimate the likely *financial impacts* of each policy scenario across retailers in neighbourhoods with different deprivation and urban/rural statuses. We measured such impacts as changes in the baseline gross profits and the proportion of retailers that would make an overall loss on tobacco sales (ie, have a negative profit).

## Methods

### Data collection and processing

#### Tobacco transactions data

The Retail Data Partnership (TRDP), a company that supplies electronic point-of-sale tills (https://shopmate.co.uk/), provided tobacco sales records from all convenience stores that used their system in Scotland. In order to capture any seasonality in tobacco sales, data were sampled from 1 week periods at each March, June, September and December between 2019 and 2022. We filtered all retailers to select those that operated for at least 5 days each week and for 2 hours on each day the store was open, yielding a final sample of 179 stores.

Sales records were defined as each item scanned on the till within each basket. Each record contained information on the *name* of tobacco product, *pack size* (amount of product sold), *gross price* (the price paid by the customer), *net price* (gross price minus value added tax), *cost price* (the price paid by the retailer to the wholesaler as reported in the price list of the wholesaler which supplied the product to each store) and *identifier of the data zone* where retailer operates in (there is a total of 6976 data zones in Scotland, representing small administrative areas comprising 500–1000 residents).^21^ Variables related to price discounts or promotions on the tobacco gross price were not considered as the free distribution of tobacco and the use of promotion coupons for retail sales is prohibited in the UK.^22^ All records were categorised into different product *types*. For this study, we retrieved data on conventional tobacco represented by four categories: ‘cigarettes’, ‘cigars’, ‘hand-rolling’ and ‘pipe’. TRDP provided pack size data in number of sticks for cigarettes and cigars and in grams for hand-rolling and pipe products. Cigarette and cigar equivalences in grams were estimated by assuming that each stick contains 0.50 and 1 g, respectively.^23^ We processed a total of 1 099 697 tobacco sales records.

All data management and analyses were conducted using R and RStudio V.3.6.1.

#### Neighbourhood indicators

We linked neighbourhood-level indicators of income deprivation and urban/rural status at data zone level to each retailer. Income deprivation was derived from the Scottish Index of Multiple Deprivation in 2020 and referred to the proportion of population receiving various forms of means tested income (eg, Income Support, Tax Credits, Guaranteed Pension Credit).^24^ We classified retailers in tertiles of income deprivation: high deprived >21%, medium deprived=11–21% and low deprived <11%. Using the 2020 Scottish Government Urban-Rural classification, we classified retailers nested in settlements larger than 3000 inhabitants as urban; otherwise, we coded them as rural.^25^

### Estimating tobacco sales volume and gross profit

We calculated weekly aggregates of tobacco sales and gross profits for each retailer and product type. We derived sales volume from the pack size variable in grams, and we defined gross profits as the difference between the net and cost price variables (see definitions above). We imputed records with missing data on pack size or net/cost price with the median values registered by the products of the same type within a given store and week, respectively, to address potential underestimations of sales and gross profits. A median of 0.72% (IQR=[0.32–2.26%]) records was imputed among stores.

We assumed that the weekly aggregates were representative of both typical tobacco sales and gross profits in each retailer in a given week within its corresponding season. Since each season has duration of 13 weeks, we estimated seasonal aggregates by multiplying weekly aggregates by 13, and then annual aggregates by adding the seasonal aggregates within each year. Finally, we calculated the average value of the annual aggregates of tobacco sales and gross profits, respectively, for the 4-year period to capture potential annual variations. Understanding any annual changes is particularly crucial, given the impact of the COVID-19 pandemic in 2020 on the retail sales and consumption of tobacco.^26^,^28^ We used a multiyear design, incorporating pre-pandemic and post-pandemic data (2019 and 2021–2022), to address potential fluctuations and obtain a robust overview of annual tobacco sales and gross profits.

To approach the level of reliance that each retailer had on the gross profits from tobacco, we calculated the proportion of whole-store profits from tobacco sales. We estimated whole-store profits using the same strategy as for calculating tobacco gross profits, accounting for all sales records conducted at each retailer beyond tobacco. The TRDP provided these data for the same weeks and format, as described in the Data collection and processing section.

### Generation of policy scenarios

Potential universal, volumetric and urban/rural fee schemes were drawn from a review of tobacco retail regulations implemented worldwide, identified through scientific and policy documents gathered from the Tobacco Control Laws Portal,^7^ and discussions with key tobacco policy advocacy stakeholders in Scotland. The universal scheme was represented by a single flat fee. The volumetric scheme was an aggregate of three components linked to the sales volume of cigarettes, cigars and hand-rolling, independently, to account for potential differences in the profit margins across tobacco types. A component targeting pipe tobacco sales was disregarded because pipe products were sold in a limited number of retailers (n=49 of 179) and accounted a small proportion of sales (n=0.1%). The urban/rural scheme consisted of a distinct component for urban and rural retailers. Online supplemental table 1 summarises the conceptualisation of the fees within each scheme type.

We defined various fee levels for each scheme using the estimations of tobacco gross profit across retailers. To assess the impact of the level at which fees were set as well as their basis, we estimated the impact of 10 different fee levels for each scheme (n=30 policy scenarios). These levels were defined in 10% increments from 10% to 100% of a theoretical maximum possible level for each scheme. Based on previous studies, we defined this maximum level as the fee level that would discourage tobacco sales from at least half of retailers.^29^,^31^ Therefore, we set the maximum level as the median gross profits from tobacco sales. For example, in the universal scheme, the fee at the 100% level would equal the median gross profits obtained by the whole sample of retailers per year. However, when considering an urban/rural scheme, the maximum level for the fee targeting urban retailers (ie, urban fee) would equal the median gross profit across retailers located in urban areas while the rural fee would equal the median gross profit across rural retailers. We used a linear equation to interpolate the full range of potential values from the modelled 10% increments.

For each of the 30 modelled scenarios, we calculated the fees that each retailer must pay and updated their gross profit from tobacco sales by subtracting the resulting fee from baseline annual gross profits.

### Measuring financial impacts of policy scenarios on retailers

The impact of each scenario was assessed using two measures. First, we calculated the changes in gross profit for each retailer and scenario, representing the proportion by which gross profits decreased in respect to the annual baseline estimates. Second, we estimated the proportion of retailers likely experiencing a loss from tobacco sales (ie, have negative annual profit). We assumed that a retailer would make a loss when the fees exceed its baseline gross profit from tobacco, potentially leading to the ceasing of tobacco sales. Furthermore, we conducted sensitivity analyses to identify retailers where fees exceeded more than 80% or 50% of gross profits, as potential concerns about the profitability of tobacco might be discussed in those cases too.

### Presentation of findings and statistical analyses

The study findings were analysed and presented following the research sequence of objectives stated in the Introduction section. We first examine outcomes about the importance of tobacco sales for retailers (ie, annual tobacco sales, gross profits and proportion of whole-store profits from tobacco). Subsequently, we present the resulting fees under the three schemes. Finally, we discuss the financial impacts of the fees on retailers, including changes in the baseline gross profits and the proportion of retailers potentially experiencing an overall loss from tobacco sales.

We grouped retailers by neighbourhood deprivation tertiles and urbanicity. We used Kruskal-Wallis non-parametric tests to assess statistically significant differences on the outcomes relating to the importance of tobacco and the financial impacts among retailers’ groups. Post-hoc comparisons (Dunn’s test) were conducted to identify which specific groups differ from each other. Additionally, we used histograms and line plots to visually explore potential changes in the shapes of the distribution of tobacco gross profits among retailers and the proportion of retailers making a loss from tobacco sales after the implementation of each policy scenario, respectively.

## Results

### Baseline differences in tobacco sales volume and gross profits

Table 1 describes baseline annual sales volume and gross profits from tobacco in our sample of retailers. The median retailer sold 247 300 g of tobacco and made £15 859 of gross profits. Gross profits were 2.29 times higher in urban neighbourhoods compared with rural areas (£18 247 vs £7638). Retailers in high deprived areas presented 1.59 times higher gross profits compared with retailers in low deprived areas (£18 403 vs £11 609). Similar differences were observed when assessing the tobacco sales volume among retailers by urbanicity and deprivation. Tobacco sales represented a median of 15.71% of the whole-store profits. These proportions were significantly higher among high-deprivation (18.14% vs low deprivation=12.94%) and urban (17.43% vs rural=11.35%) retailers.

**Table 1:** Description of baseline sales and gross profits from tobacco among retailers by area types (median values and 95% CI in brackets)

Outcomes	All retailers	Area income deprivation			Area urban/rural	
		High deprived	Medium	Low deprived	Urban	Rural
Number of retailers	179	60	57	62	137	42
Median gross profit/retailer (£/year) (95% CI)						
All tobacco	15 859.0 (2992.0 to 40 193.8)	18 403.3 (5047.0 to 43 503.4)	17 693.9 (5322.0 to 36 579.8)	11 608.7 (2186.7 to 32 258.3)**	18 247.4 (4202.1 to 40 998.6)**	7637.9 (2007.2 to 24 593.0)**
Cigarettes (per 1000 sticks)^1^	33.24 (23.22 to 67.47)	33.41 (22.80 to 52.47)	32.71 (23.59 to 66.19)	33.34 (24.14 to 69.42)	33.49 (23.78 to 66.19)	32.59 (23.16 to 67.61)
Cigars (per 1000 g)	55.63 (31.93 to 157.08)	43.91 (31.80 to 137.22)	56.71 (33.14 to 124.23)	67.60 (33.61 to 237.77)**	51.91 (31.82 to 153.08)**	67.08 (37.24 to 167.48)**
Hand-rolling (per 1000 g)	40.41 (30.83 to 69.14)	40.63 (31.13 to 67.93)	39.71 (30.80 to 64.38)	41.50 (34.01 to 74.55)	41.08 (31.44 to 69.43)*	38.92 (30.12 to 65.61)*
Median sales volume (per year) (95% CI)						
All tobacco (per 1000 g)	247.30 (47.61 to 613.27)	309.23 (80.28 to 667.45)	260.45 (90.82 to 612.02)	172.78 (32.40 to 461.05)**	280.21 (57.70 to 649.02)**	146.50 (29.14 to 506.21)**
Cigarettes (per 1000 sticks)^1^	336.71 (54.40 to 899.27)	424.88 (98.98 to 939.98)	331.10 (114.96 to 903.58)	239.57 (38.45 to 699.74)**	396.68 (95.15 to 932.03)**	174.70 (36.91 to 704.61)**
Cigars (per 1000 g)	3.36 (0.00 to 14.25)	3.85 (0.37 to 10.19)	3.74 (0.60 to 20.10)	3.01 (0.17 to 10.77)	3.80 (0.34 to 14.56)**	2.55 (0.065 to 12.09)**
Hand-rolling (per 1000 g)	70.20 (10.83 to 228.12)	96.57 (17.39 to 235.82)	83.53 (17.65 to 232.47)	48.62 (10.36 to 167.72)**	83.04 (14.41 to 232.47)**	48.62 (10.58 to 161.92)**
Median proportion of tobacco profits over the whole-store profits (95% CI)	15.71 (6.16 to 46.98)	18.14 (10.93 to 55.98)	17.31 (10.32 to 30.99)	12.94 (3.96 to 45.07)**	17.43 (9.58 to 60.59)**	11.35 (3.59 to 20.03)**

*indicates statistically significant differences among area types: p<0.05; and **indicates statistically significant differences among area types: p<0.01.

Post-hoc (Dunn’s test) results are shown within area income deprivation groups.

1We assumed that one cigarette stick contains 0.50 g of tobacco. This way, 1000 sticks equate to 500 g of tobacco.

### Resulting fees for policy scenarios

**Figure 1:**
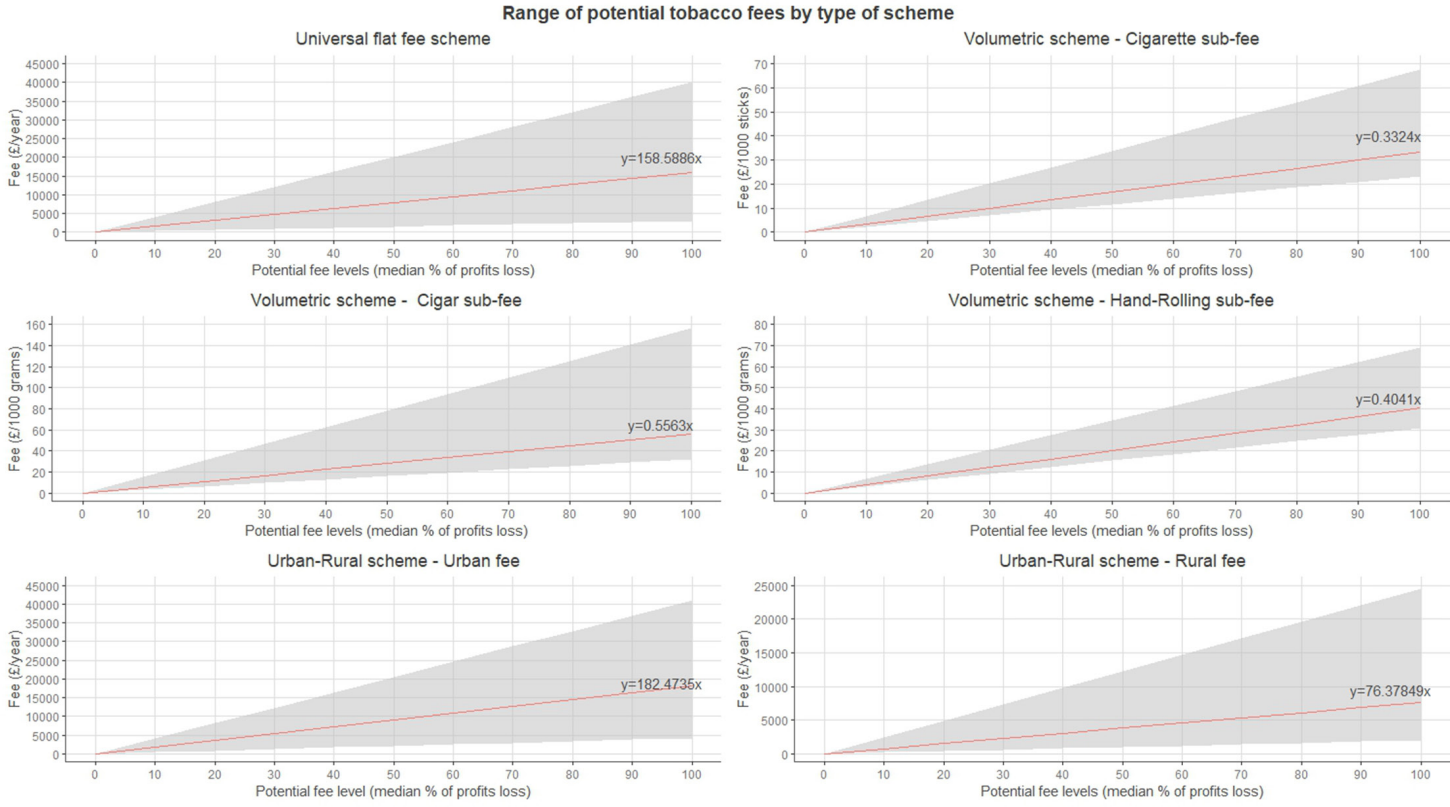
Range of potential tobacco fees by type of scheme. Shadowed areas within each graph represent the 95% CI for the predicted values for the fees. Data source: authors from The Retail Data Partnership, 2023.

The potential fees ranged from £0 (no fee/baseline scenario) to £15 859/year (maximum fee level) for universal scheme scenarios; to £33.24/1000 cigarette sticks, £55.63/1000 g of cigars and £40.41/1000 g of hand-rolling for volumetric scheme scenarios; and to £18 247/year and £7638/year for retailers in urban and rural areas, respectively, for the urban/rural scheme scenarios. These fees are displayed in figure 1. The fees’ 95% CIs, indicating the variation resulting from the uncertainty around the median gross profits across retailers, were wider when considering higher fee levels. Online supplemental table 2 standardised these fees in pounds/year units across our sample of retailers, enabling direct comparisons between scheme types and levels.

### Financial impacts of fees: potential changes in tobacco gross profits

**Figure 2:**
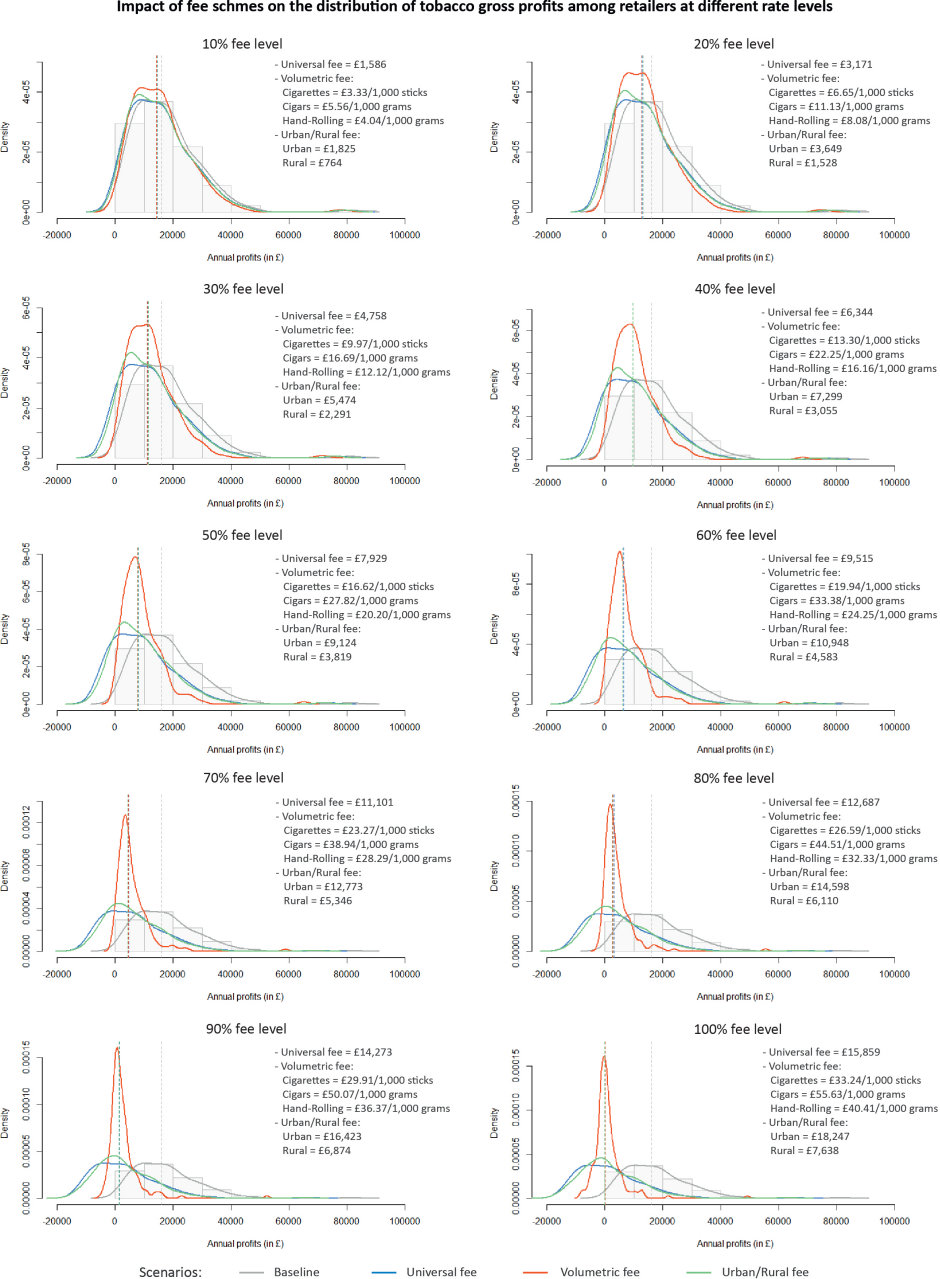
Impact of fee schemes on the distribution of tobacco gross profits among retailers at different rate levels. Each graph includes the following set of elements: (1) the original histogram of the distribution of tobacco profits in the baseline scenario (no fees implemented) represented by light grey bars of 10 000 bins; (2) continuous lines showing the curve of density of histograms representing the distribution of tobacco gross profits for each policy scenario (baseline, universal fee, volumetric fee and urban/rural fee); and (3) dashed lines representing the median values for each histogram and policy scenario. The curve of density was calculated using Kernel Density Estimations. Authorship and data source: authors from The Retail Data Partnership, 2023.

Figure 2 compares overall modelled changes in the distribution of tobacco gross profits among retailers after the introduction of universal, volumetric and urban/rural fees at different levels. The baseline distribution of tobacco gross profits showed a positive skewed shape. The changes in the distribution for the three scheme types were relatively modest at low fee levels, but became more pronounced as higher fee levels were introduced. Moreover, each scheme type exhibited a distinct pattern of change after its introduction, which was observed independently of the fee levels’ effect on the magnitude of changes. Universal fees shifted the baseline distribution to lower values of profits in proportion to the fee and preserved the positive skewed shape in the distribution. Urban/rural fee schemes followed a similar pattern, but distributions showed a slight squeeze effect due to lower density of retailers making negative profits and higher concentration of stores below the median. The squeeze effect is more evident under a volumetric scheme, which moved retailers from high to median values of profits and minimised the density of retailers making negative profits.

Table 2 shows differences in the median percentage change in gross profits across retailers by geography. Retailers located in low-deprivation and rural areas showed the greatest median gross profit reduction under a universal scheme. For example, universal fees at the 10% level reduced gross profit by 13.66% among retailers in low deprived areas, compared with 8.62% and 8.96% reductions in high and medium deprived ones (p<0.01). These trends were consistent across different fee levels. Volumetric fees yielded similar median percentage changes in gross profits across retailers by geography with no statistically significant differences. Urban/rural fees led to similar median profit changes for retailers in urban and rural areas, but higher profit reductions for retailers in low deprived areas (vs high/medium deprived). However, these differences were not statistically significant.

**Table 2:** Median percentage change in gross profit across retailers by area types (IQR values in brackets)

Fee level	Scheme	All	Area types: income deprivation				Area types: urban status		
			High deprived	Medium deprived	Low deprived	P value	Urban	Rural	P value
10%	Universal	−10.00% (−6.87, –17.64)	−8.62% (−5.51, –12.27)	−8.96% (−6.24, –15.46)	−13.66% (−8.63, –25.87)	<0.00	−8.69% (−6.21, –13.93)	−20.76% (−10.82, –29.34)	<0.00
	Volumetric	−9.89% (−8.49, –11.20)	−9.86% (−8.35, –11.36)	−10.07% (−8.83, –11.33)	−9.78% (−8.13, –10.99)	0.67	−9.85% (−8.35, –10.99)	−10.21% (−9.01, –12.05)	0.13
	Urban/rural	−10.00% (−6.65, –15.66)	−9.38% (−6.10, –13.59)	−9.82% (−6.73, –14.93)	−12.11% (−8.85, –17.82)	0.09	−10.00% (−7.15, –16.03)	−10.00% (−5.21,–14.13)	0.21
20%	Universal	−20.00% (−13.73, –35.29)	−17.23% (−11.02, –24.54)	−17.93% (−12.48, –30.92)	−27.32% (−17.25, –51.73)	<0.00	−17.38% (−12.44, –27.87)	−41.53% (−21.64, –58.58)	<0.00
	Volumetric	−19.79% (−16.97, –22.40)	−19.72% (−16.70, –22.72)	−20.14% (−17.6, –22.66)	−19.59% (−16.25, –21.98)	0.67	−19.70% (−16.70, –21.99)	−20.42% (−18.03, –24.11)	0.13
	Urban/rural	−20.00% (−13.30, –31.33)	−18.75% (−12.19, –27.18)	−19.64% (−13.46, –29.86)	−24.22% (−17.70, –35.63)	0.09	−20.00% (−14.31, –32.06)	−20.00% (−10.42, –28.26)	0.21
30%	Universal	−30.00% (−20.60, –52.93)	−25.85% (−16.53, –36.80)	−26.89% (−18.71, –46.38)	−40.98% (−25.88, –77.59)	<0.00	−26.07% (−18.65, –41.80)	−62.29% (−32.46, –88.02)	<0.00
	Volumetric	−29.68% (−25.45, –33.60)	−29.58% (−25.06, –34.08)	−30.22% (−26.49, –33.99)	−29.35% (−24.38, –32.97)	0.67	−29.55% (−25.05, –32.98)	−30.63% (−27.04, –36.16)	0.13
	Urban/rural	−30.00% (−19.95, –46.99)	−28.13% (−18.28, –40.76)	−29.46% (−20.19, –44.80)	−36.33% (−26.55, –53.44)	0.09	−30.00% (−21.46, –48.09)	−30.00% (−15.63, –42.39)	0.21
40%	Universal	−40.00% (−27.47, –70.58)	−34.47% (−22.04, 49.07)	−35.85% (−24.94, –61.85)	−54.65% (−34.50, –103.46)	<0.00	−34.76% (−24.87, –55.73)	−83.05% (−43.28, –117.36)	<0.00
	Volumetric	−39.57% (−33.94, –44.80)	−39.4430% (−33.41, –45.44)	−40.29% (−35.32, –45.32)	−39.13% (−32.51, –43.95)	0.67	−39.40% (−33.40, –43.97)	−40.84% (−36.06, –48.21)	0.13
	Urban/rural	−40.00% (−26.60, –62.65)	−37.50% (−24.38, –54.35)	−39.28% (−26.92, –59.73)	−48.44% (−35.40, –71.26)	0.09	−40.00% (−28.62, –64.13)	−40.00% (−20.85, –56.52)	0.21
50%	Universal	−50.00% (−34.33, –88.22)	−43.09% (−27.55, –61.34)	−44.81% (−31.18, –77.31)	−68.31% (−43.13, –129.32)	<0.00	−43.46% (−31.09, –69.67)	−103.82% (−54.10, –146.70)	<0.00
	Volumetric	−49.46% (−42.42, –56.00)	−49.30% (−41.76, –56.80)	−50.36% (−44.15, –56.65)	−48.92% (−40.64, –54.94)	0.67	−49.25% (−41.75, –54.97)	−51.05% (−45.07, –60.26)	0.13
	Urban/rural	−50.00% (−33.25, –78.32)	−46.88% (−30.47, –67.94)	−49.11% (−33.66, –74.66)	−60.55% (−44.25, –89.07)	0.09	−50.00% (−35.77, –80.16)	−50.00% (−26.05, –70.65)	0.21
60%	Universal	−60.00% (−41.20, –105.86)	−51.70% (−33.06, –73.61)	−53.78% (−37.41, –92.77)	−81.97% (−51.75, –155.19)	<0.00	−52.15% (−37.31, –83.60)	−124.58% (−64.92, –176.04)	<0.00
	Volumetric	−59.35% (−50.91, –67.20)	−59.16% (−50.11, –68.16)	−60.43% (−52.98, –67.98)	−58.70% (−48.76, –65.93)	0.67	−59.10% (−50.10, –65.96)	−61.26% (−54.08, –72.32)	0.13
	Urban/rural	−60.00% (−39.90, –93.98)	−56.26% (−36.57, –81.53)	−58.93% (−40.39, –89.59)	−72.66% (−53.10, –106.89)	0.09	−60.00% (−42.92, –96.19)	−60.00% (−31.27, –84.78)	0.21
70%	Universal	−70.00% (−48.07, –123.51)	−60.32% (−38.57, –85.88)	−62.74% (−43.65, –108.23)	−95.63% (−60.38, –181.05)	<0.00	−60.84% (−43.52, –97.53)	−145.34% (−75.74, –205.38)	<0.00
	Volumetric	−69.25% (−59.39, –78.40)	−69.02% (−58.46, –79.52)	−70.50% (−61.81, –79.31)	−68.48% (−56.89, –76.92)	0.67	−68.95% (−58.45, –76.95)	−71.63% (−63.10, –84.37)	0.13
	Urban/rural	−70.00% (−46.54, –109.64)	−65.63% (−42.66, –95.11)	−68.75% (−47.12, –104.52)	−84.77% (−61.95, –124.70)	0.09	−70.00% (−50.08, –112.22)	−70.00% (−36.48, –98.91)	0.21
80%	Universal	−80.00% (−54.93, –141.15)	−68.94% (−44.08, –98.14)	−71.70% (−49.88, –123.69)	−109.29% (−69.00, –206.92)	<0.00	−69.53% (−49.74, –111.46)	−166.11% (−86.56, –234.72)	<0.00
	Volumetric	−79.14% (−67.88, –89.60)	−78.88% (−66.81, –90.88)	−80.58% (−70.64, –90.64)	−78.27% (−65.02, –87.91)	0.67	−78.80% (−66.80, –87.95)	−81.69% (−72.11, –96.42)	0.13
	Urban/rural	−80.00% (−53.19, –125.31)	−75.01% (−48.76, –108.70)	−78.57% (−53.85, –119.45)	−96.88% (−70.80, –142.52)	0.09	−80.00% (−57.23, –128.25)	−80.00% (−41.69, –113.04)	0.21
90%	Universal	−90.00% (−61.80, –158.80)	−77.56% (−49.60, –110.41)	−80.67% (−56.12, –139.15)	−122.95% (−77.63, –232.78)	<0.00	−78.22% (−55.96, –125.40)	−186.87% (−97.38, –264.06)	<0.00
	Volumetric	−89.03% (−76.36, –100.80)	−88.74% (−75.17, –102.24)	−90.65% (−79.47, –101.94)	−88.05% (−73.14, –98.90)	0.67	−88.65% (−75.15, –98.94)	−91.90% (−81.13, –108.47)	0.13
	Urban/rural	−90.00% (−59.84, –140.97)	−84.39% (−54.85, –122.29)	−88.39% (−60.58, –134.39)	−108.99% (−79.64, –160.33)	0.09	−90.00% (−64.39, –144.28)	−90.00% (−46.90, –127.17)	0.21
100%	Universal	−100.00% (−68.66, –176.44)	−86.17% (−55.11, –122.68)	−89.63% (−62.36, –154.61)	−136.61% (−86.25, –258.65)	<0.00	−86.91% (−62.18, –139.33)	−207.64% (−108.20, –293.40)	<0.00
	Volumetric	−98.92% (−84.85, –112.01)	−98.59% (−83.52, –113.61)	−100.72% (−88.30, –113.30)	−97.83% (−81.27, –109.89)	0.67	−98.50% (−83.51, –109.93)	−102.11% (−90.14, –120.53)	0.13
	Urban/rural	−100.00% (−66.49, –156.63)	−93.76% (−60.95, –135.88)	−98.21% (−67.31, –149.32)	−121.10% (−88.49, –178.15)	0.09	−100.00% (−71.54, –160.31)	−100.00% (−52.11, –141.31)	0.21

Values over 100% indicate that fees would exceed the gross profit from tobacco sales.

Findings from table 2 are complemented by online supplemental figure 1 which provides a visual analysis of the histograms filtered by geography.

### Financial impacts of fees: proportion of retailers likely to cease tobacco sales

**Figure 3:**
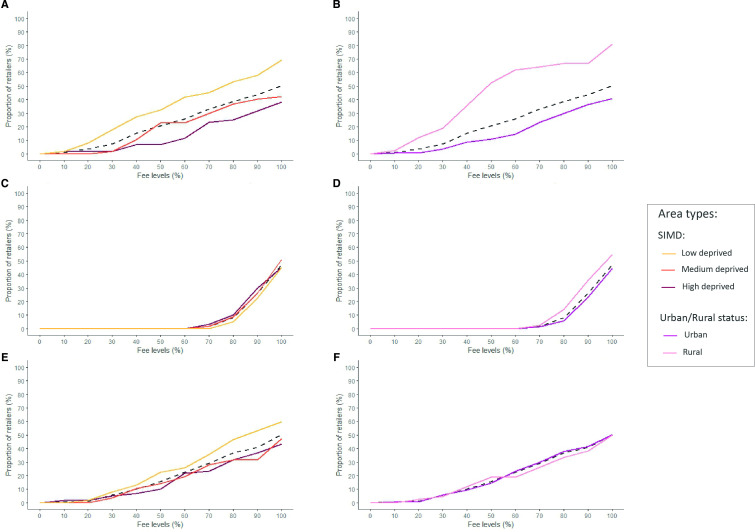
Estimated proportion of retailers potentially ceasing tobacco sales. (A) Universal fee scenarios: impact on retailers by SIMD. (B) Universal fee scenarios: impact on retailers by urban/rural status. (C) Volumetric fee scenarios: impact on retailers by SIMD. (D) Volumetric fee scenarios: impact on retailers by urban/rural status. (E) Urban/rural fee scenarios: impact on retailers by SIMD. (F) Urban/rural fee scenarios: impact on retailers by urban/rural status. Authorship and data source: authors from The Retail Data Partnership, 2023. SIMD, Scottish Index of Multiple Deprivation.

Figure 3 estimates the proportion of retailers likely making a loss and therefore expected to cease tobacco sales after the introduction of each policy scenario. Universal fees consistently led to the highest proportions at all fee levels compared with other schemes (eg, 7.26% at 30% fee level vs volumetric=0% vs urban/rural=5.59%). These proportions were higher for retailers in low-deprivation (eg, 17.14% at 30% fee level vs 1.67% high-deprivation) and rural (eg, 19.05% at 30% fee level vs 3.65% urban) areas. These differences among retailer types increased when scenarios with high fee levels were considered.

In contrast, no retailers were expected to cease tobacco sales when volumetric fees were implemented below the 60% level. However, when applying fees higher than 60% level, the proportions of retailers ceasing tobacco sales were slightly higher in rural (vs urban) areas, but they were relatively similar among neighbourhood deprivation tertiles.

Urban/rural fees led to moderate overall proportions of retailers ceasing tobacco sales. Proportions were higher among low-deprivation retailers (eg, 8.06% at 30% fee level vs 5.00% high-deprivation). However, these differences were lower than those observed in the universal scenarios; and they even disappeared by urbanicity.

Results from sensitivity analyses varying the point at which retailers might consider ceasing tobacco sales described similar patterns (see online supplemental figures 2 and 3).

## Discussion

Using electronic transaction data on 1 099 697 tobacco sales records in 179 convenience stores across Scotland, this paper estimated the financial impacts on retailers from the introduction of different licencing fees to regulate tobacco sales. We assessed 30 policy scenarios considering a wide range of fee levels within universal, volumetric and urban/rural schemes, which yielded disparate financial impacts by geography.

Universal flat fees resulted in significantly higher profit reductions in retailers selling fewer tobacco products, predominantly in low-deprivation and rural areas, and favoured retailers in urban and high-deprivation areas with higher sales and gross profits from tobacco. This approach preserved the baseline absolute differences in tobacco gross profits among retailers and achieved the highest proportion of retailers likely to cease tobacco sales, mostly unevenly located in rural and low-deprivation areas. In contrast, the volumetric scheme levied the highest fees to retailers selling more tobacco and the lowest fees to retailers with lower sales volume, achieving equal profit reductions among retailers. However, we should envisage that the affordability of paying volumetric fees may vary among retailers. Retailers with greater sales volume, more diversified business models or located in more profitable neighbourhoods (ie, urban areas) might have greater financial resources to cope with a given loss of 10% of their profits compared with other retailers. Nevertheless, volumetric fees resulted in the lowest proportion of retailers likely ceasing tobacco sales compared with other schemes. The urban/rural scheme positively impacted rural retailers with lower sales and gross profit from tobacco by imposing the lowest flat fees. However, retailers located in less deprived areas might experience greater reductions in gross profits as compared with retailers in high-deprivation areas.

When defining a new licencing fee scheme, policymakers should acknowledge existing spatial differences in the distribution of tobacco sales and gross profits among retailers to prevent any geographical bias in the policy’s impacts and effectiveness. In this context, it is essential to recognise that tobacco retailers in Scotland do not just sell tobacco. Most of them are small convenience stores who may also provide essential services to population and can serve as a community hub for social gathering.^32 33^ The role of small convenience stores may be particularly important in rural or high-deprivation areas where they may be one of few retailers in the community. Small retailers in these particular areas might be more sensitive to profit reductions and future licence fee schemes should be designed to avoid undermining their business models. This geographical equity lens is crucial to prevent other critical social and economic problems associated with potential retailer shutdowns in these communities.

However, protecting the retailer sustainability should not imply maintaining unfettered geographical access to tobacco, especially considering the associated risks to public health. Indeed, licencing fee schemes are ultimately intended to restrict the geographical availability and reduce the use of tobacco products, leading to the reduction of tobacco-related morbidity and mortality, especially for those groups and neighbourhoods disproportionately exposed to tobacco. The design and implementation of policies that maximise the tobacco outlet availability reduction effect to mitigate smoking-related harms on population health without threatening retail viability would significantly contribute to the sustainability of communities in Scotland and globally. One possible strategy might include a modest flat fee, which varies between urban and rural areas (ie, urban/rural scheme), along with an additional moderate volumetric fee in Scotland.

The magnitude of the financial impacts of schemes on retailers was significantly modulated by the fee level. Fees achieving a 50% reduction of retailers selling tobacco were substantially higher than those currently implemented in other countries (eg, universal fee in Scotland=£15 859/year, vs universal fee in Finland=€500/year) and would likely face opposition from the tobacco industry and the retail sector. However, previous studies demonstrate the limited impact of existing licence fees in reducing tobacco retail availability^34^ and argue for higher fee levels.^35^ Applying fees similar to those implemented elsewhere, for instance, in New Brunswick (Canada), Western Australia and Finland, would lead to a reduction of 0.18%, 1.02% or 2.75% in median profits loss in our sample of retailers in Scotland, respectively (see equations in figure 1). These low-level fees might not effectively discourage retailers from selling tobacco, as they might introduce unsubstantial changes in retailers’ gross profits. Equally, it is important to consider that higher fees may impact large retailers (eg, supermarkets, etc) less due to having a more diversified range of products and services to absorb the potential fee costs than small retailers.^36^

Our findings underscore the importance of future tobacco retail reduction policies to provide economically viable alternatives to support all types of retailers across different geographies to diversify their business models away from tobacco, aligning with the Article 17 of the WHO Framework Convention on Tobacco Control.^8 37^ Such measures could include temporary government payments or other types of financial or tax incentives to give up tobacco sales.^8 36 38^ Evidence from elsewhere suggests that stores may benefit from ceasing tobacco sales with such benefits including enhancement of the store’s image and improved public relations.^39^

This study presents several strengths. Our results shed light on the intricate relationship between fee-based policies and retailers’ financial outcomes, which can inform evidence-based decisions for future retail licencing strategies. For example, the Scottish legislation requires the development of a Business Regulatory Impact Assessment (BRIA) to anticipate costs, benefits and risks of a proposed legislation prior to its introduction.^40^ This work provides valuable evidence to the BRIA on the feasibility of implementing various forms of licencing schemes and their impacts on retailers’ profits and the broader economic landscape in their local communities. The presented results can foresee public health issues derived from licencing policies resulting on a high availability of tobacco products or small retailers’ shutdowns across communities, which are relevant to the BRIA, as well. Similarly, researchers and policymakers producing evidence in other countries aiming to introduce licencing fee schemes to regulate tobacco sales might benefit from the methods and findings presented.

Further, our estimations are derived from detailed electronic transaction data, which are rarely available for research. This data source allowed us to enhance our understanding of tobacco sales’ significance for retailers and explore potential licencing strategies. The involvement of stakeholders in choosing these strategies ensured our research aligned with specific policy decision-making needs.

However, some limitations should be acknowledged in this study. The Tobacco Advertising and Promotion Act 2002 bans promotions, free gifts and coupons for retail sales of tobacco in UK but this does not apply to wholesaler sales.^22^ Although it is unlikely that wholesalers provide free tobacco products because their margin profits from tobacco are too low in the UK, they may offer vouchers to buy or get free stock of other non-tobacco products via tobacco company representatives.^41 42^ These specific strategies can effectively reduce the cost of tobacco for retailers, increasing their profits from tobacco sales. TRDP data did not cover promotions from the wholesalers to the retailers and the extent of these strategies could not be explored within our dataset. Furthermore, our decision of using gross profit as financial indicator may overestimate the profitability of tobacco for smaller retailers as it does not account for the retailers’ overheads (ie, expenses associated with running a business such as rent, electricity bills, equipment, etc). However, we estimated a flexible range of fee levels and developed sensitivity analyses varying the rules through which retailers were considered to likely stop tobacco sales.

The values resulting from the highest fee levels presented the widest CIs and should be interpreted cautiously. Future research might employ approaches such as Bayesian methods to obtain more robust predictions.

Another potential limitation is that TRDP offers data from an opportunity sample of retailers which might not be representative of the socioeconomic spectrum of neighbourhoods across Scotland. Indeed, the number of retailers working with TRDP is slightly skewed to urban areas (137 urban vs 42 rural retailers) and highly deprived neighbourhoods (cut-off values for tertiles of income deprivation in the whole of Scotland were 6% and 15%, while in our sample, these were 11% and 21%, see the Data collection and processing section). However, this skewed geographical distribution likely reflects the inherent spatial pattern of the location of convenience stores. Evidence in the UK noted that 63% of convenience stores were located in urban areas (vs 37% in rural neighbourhoods).^43^ Previous research in Scotland also highlighted higher concentrations of convenience stores selling tobacco in the most deprived neighbourhoods.^44^ Recognising the spatial patterning of the distribution of different types of tobacco retailers is key to understand which geographies and communities will be more affected by the implementation of different licencing strategies.

Our study focused on convenience stores, and information from other types of tobacco retailers (particularly larger supermarkets) was not available. Nevertheless, convenience stores represented 74.54% of tobacco retailers in Scotland^45^ and accounted for 55–60% of total cigarette sales in the UK, being the most common retail source of tobacco product purchases.^46^ Furthermore, large retailers may experience fewer challenges in adapting their businesses to policies regulating tobacco sales.^36^

The presented analyses do not capture the dynamic nature of the retail tobacco environment. It is likely that tobacco industry and retailers would undertake various actions to mitigate potential profit loss resulting from the introduction of licencing fees. This may include implementing price-discriminating strategies to pass to customers some or all of the increased costs of selling tobacco.^47 48^ Further research could usefully employ a system dynamics analytical approach, such as Agent-Based Modelling to assess different fee scheme scenarios and the subsequent impact on smoking prevalence and health-related harms.^3149^,^51^

The implementation of tobacco licence fees is likely to face important challenges, which also deserve further investigation. Licencing schemes require the development of an organisational structure to administer the fees and monitor their compliance. Volumetric fees pose additional complexities when compared with other schemes as they need to be individually calculated for each retailer. Retailers may also be hesitant to disclose information about their sales, further complicating evaluation and implementation.^1 52^ Future qualitative research may anticipate potential objections against licence fees from retailers, political entities or the public. However, public support for tobacco availability reduction policies is strong in Scotland.^53^

## Conclusion

The introduction of tobacco licence fees offers a potential opportunity for reducing the availability of tobacco retailers. However, understanding the trade-off between the benefits of these policies in reducing the availability of tobacco and its economic impact on retailers and communities is crucial. The likely impact of a tobacco licence fee is sensitive to the type of licence scheme implemented, the level at which fees are set and the retailers’ location in relation to neighbourhood deprivation and rurality. Future tobacco retail reduction strategies should acknowledge the important role of retailers sustaining communities in many areas and offer additional support for an economically viable transition away from tobacco products.

## Supplementary material

10.1136/tc-2023-058342 online supplemental file 1

## Data Availability

Data are available upon reasonable request. Data may be obtained from a third party and are not publicly available. Data on tobacco transactions must be requested from The Retail Data Partnership. Data on neighbourhood income deprivation and urban status are already available from the Scottish Government.
