# Supplementary material for: Geographical differences in the financial impacts of different forms of tobacco licence fees on small retailers in Scotland
Source: Tob Control. Author manuscript; Available in PMC 2026 Jul 31. (PMC12168086; doi:10.1136/tc-2023-058342)

APPENDIX – SUPPLEMENTARY MATERIAL

Table S1. Conceptualisation of fee schemes and policy scenarios.

Scheme type	Components (Y) [1]	Unit cost	Eligible retailers	Maximum rate level (Max)[2]	Equation to estimate components (Y)	Final Fee
**Universal flat fee**	Universal fee (Y_{univ})	Pounds (£) per year	All retailers	Max_{univ} = Median annual gross profits across all retailers in the dataset (2019-2022)	Y_{univ}= (Max_{univ} * X) / 100	Y_{univ}
**Volumetric fee**	Cigarette sub-fee (Y_{cigs})	Pounds (£) per each 1,000 sticks sold/year[3]	All retailers selling cigarettes	Max_{cigs} = Median gross margin profits obtained from the sales of 1,000 cigarette sticks across all retailers in the dataset (2019-2022)	Y_{cigs}= (Max_{cigs} * X) / 100	[(Y_{cigs} * $S_{cigs\ i}$)/1000] + [(Y_{cigar} * $S_{cigar\ i}$)/1000] + [(Y_{hr} * $S_{hr\ i}$)/1000]
	Cigar sub-fee (Y_{cigar})	Pounds (£) per each 1,000 grams sold/year[3]	All retailers selling cigars	Max_{cigar} = Median gross margin profits obtained from the sales of 1,000 grams of cigars across all retailers in the dataset (2019-2022)	Y_{cigar}= (Max_{cigar} * X) / 100	
	Hand rolling sub-fee (Y_{hr})	Pounds (£) per each 1,000 grams sold/year[3]	All retailers selling hand rolling tobacco	Max_{hr} = Median gross margin profits obtained from the sales of 1,000 grams of hand-rolling tobacco across all retailers in the dataset (2019-2022)	Y_{hr}= (Max_{hr} * X) / 100	
**Urban/Rural fee**	Urban fee (Y_{ur})	Pounds (£) per year	Urban retailers	Max_{ur} = Median annual gross profits across urban retailers in the dataset (2019-2022)	Y_{ur}= (Max_{ur} * X) / 100	Y_{ur}
	Rural fee (Y_{ru})	Pounds (£) per year	Rural retailers	Max_{ru} = Median annual gross profits across rural retailers in the dataset (2019-2022)	Y_{ru}= (Max_{ru} * X) / 100	Y_{ru}

[1] Notation in equations:

- *Y* represents the fee components (i.e., amount of money to pay for each component within each fee scheme).
- *X* represents the rate level in percent scale; $X \in \mathbb{N} = [0, 100]$
- *Max* is the amount of money representing the highest possible rate for each fee component (100% rate level, i.e., X=100).
- *S* represents the tobacco sales volume in a given retailer *i*, defined as 1) no. cigarette sticks sold (*Scigs i*); 2) grams of cigar sold (*Scigar i*); or 3) grams of hand-rolling sold (*Shr i*).

[2] Rationale for maximum rate: this may make tobacco sales unprofitable for 50% of retailers in Scotland.

[3] These measurement units were selected for consistency with the ones used by the UK Government for tobacco taxation. See: UK HM Revenue & Customs. Excise Notice 476: Tobacco product Duty. 2021.https://www.gov.uk/government/publications/excise-notice-476-tobacco-products-duty (accessed 17 Nov 2022).

Table S2. Resulted fees standardised in pounds per year across our sample of retailers by scheme type and level.

Potential Fee Levels	Universal Fee (All retailers)	Volumetric Fee (All retailers – Median values and IQR)	Urban/Rural Fee	
			Urban Retailers	Rural Retailers
10%	£1,585.89	£1,461.50 [898.60 – 2,224.50]	£1,824.74	£763.78
20%	£3,171.77	£2,922.90 [1,797.30 – 4,449.00]	£3,649.47	£1,527.57
30%	£4,757.66	£4,384.40 [2,696.00 – 6,6673.50]	£5,474.21	£2,291.35
40%	£6,343.55	£5,845.90 [3,594.60 – 8,898.10]	£7,298.94	£3,055.14
50%	£7,929.43	£7,307.40 [4,493.30 – 11,122.60]	£9,123.68	£3,818.92
60%	£9,515.32	£8,768.80 [5,391.90 – 13,347.10]	£10,948.41	£4,582.71
70%	£11,101.20	£10,230.30 [6,290.60 – 15,571.60]	£12,773.15	£5,346.49
80%	£12,687.09	£11,692.00 [7,189.00 – 17,796.00]	£14,597.88	£6,110.28
90%	£14,272.98	£13,153.00 [8,088.00 – 20,021.00]	£16,422.62	£6,874.06
100%	£15,858.86	£14,615.00 [8,986.00 – 22,245.00]	£18,247.35	£7,637.85

Figure S1. Impact of fee schemes at 50% level on the distribution of tobacco gross profits among retailers at different geographies.

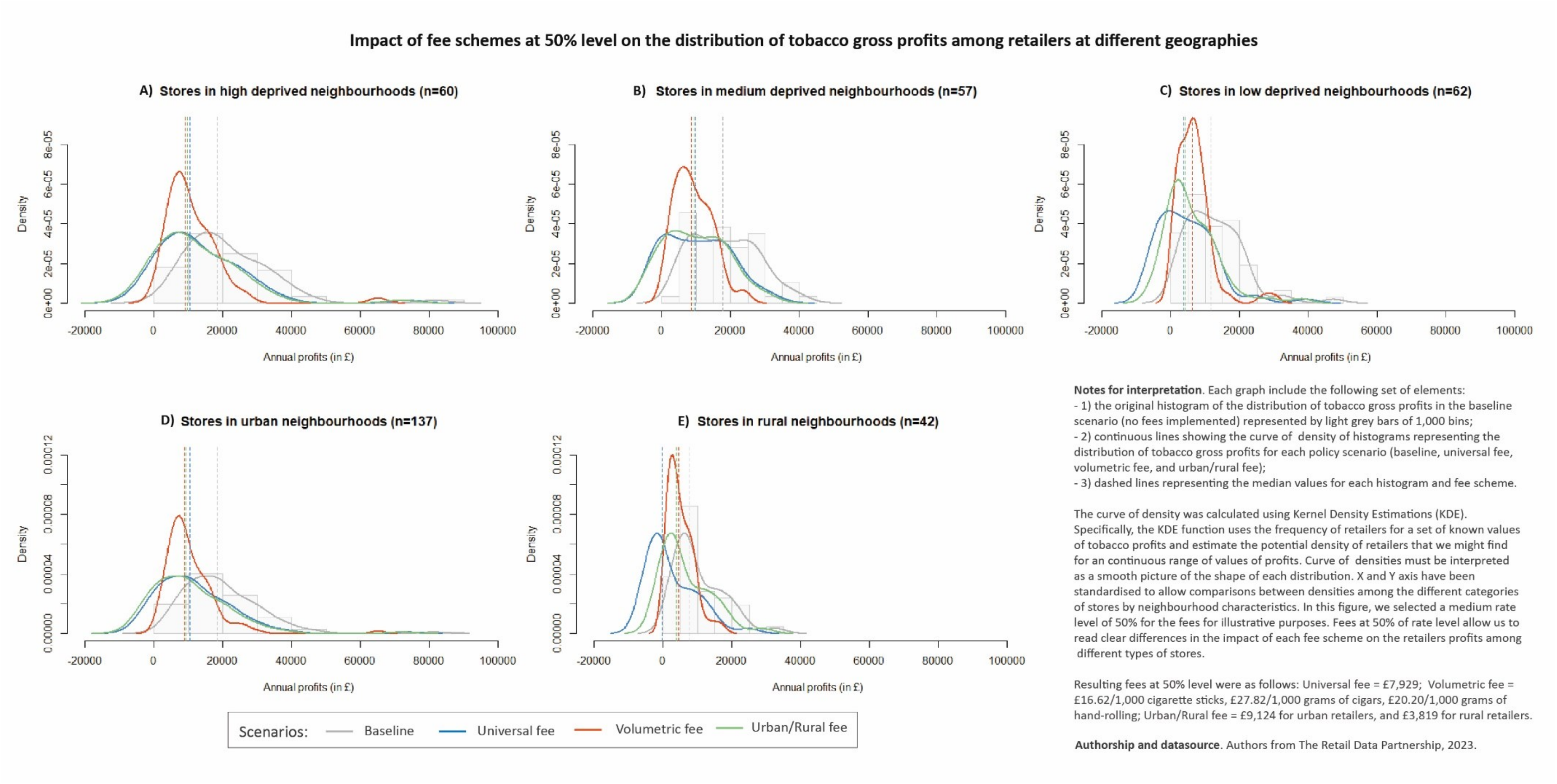

Footnote: This level was chosen for illustrative purposes and it may lead to substantially higher fees than the ones implemented worldwide.

Figure S2. Estimated proportion of retailers potentially ceasing tobacco sales (sensitivity analysis 1).

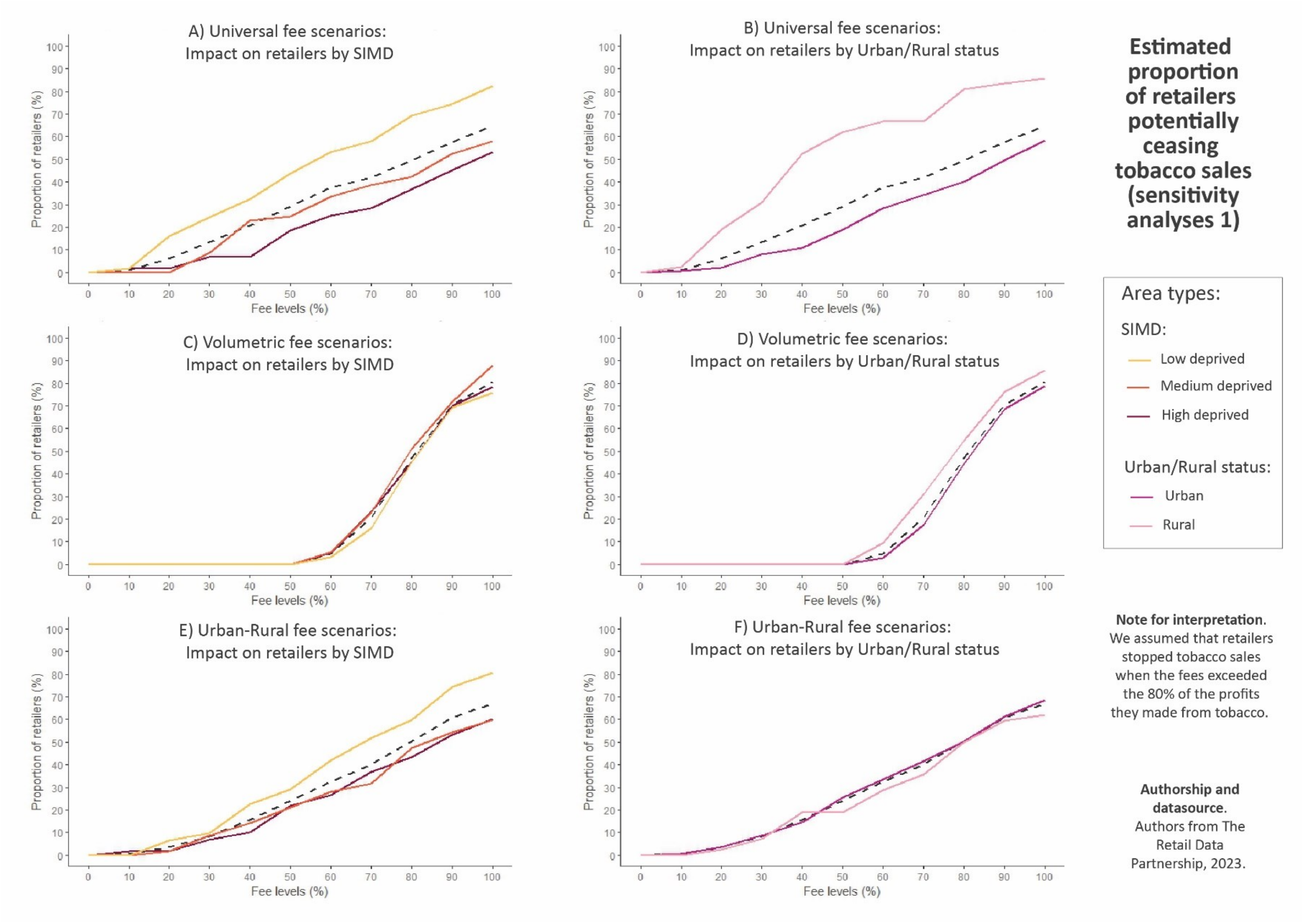

Figure S3. Estimated proportion of retailers potentially ceasing tobacco sales (sensitivity analysis 2).

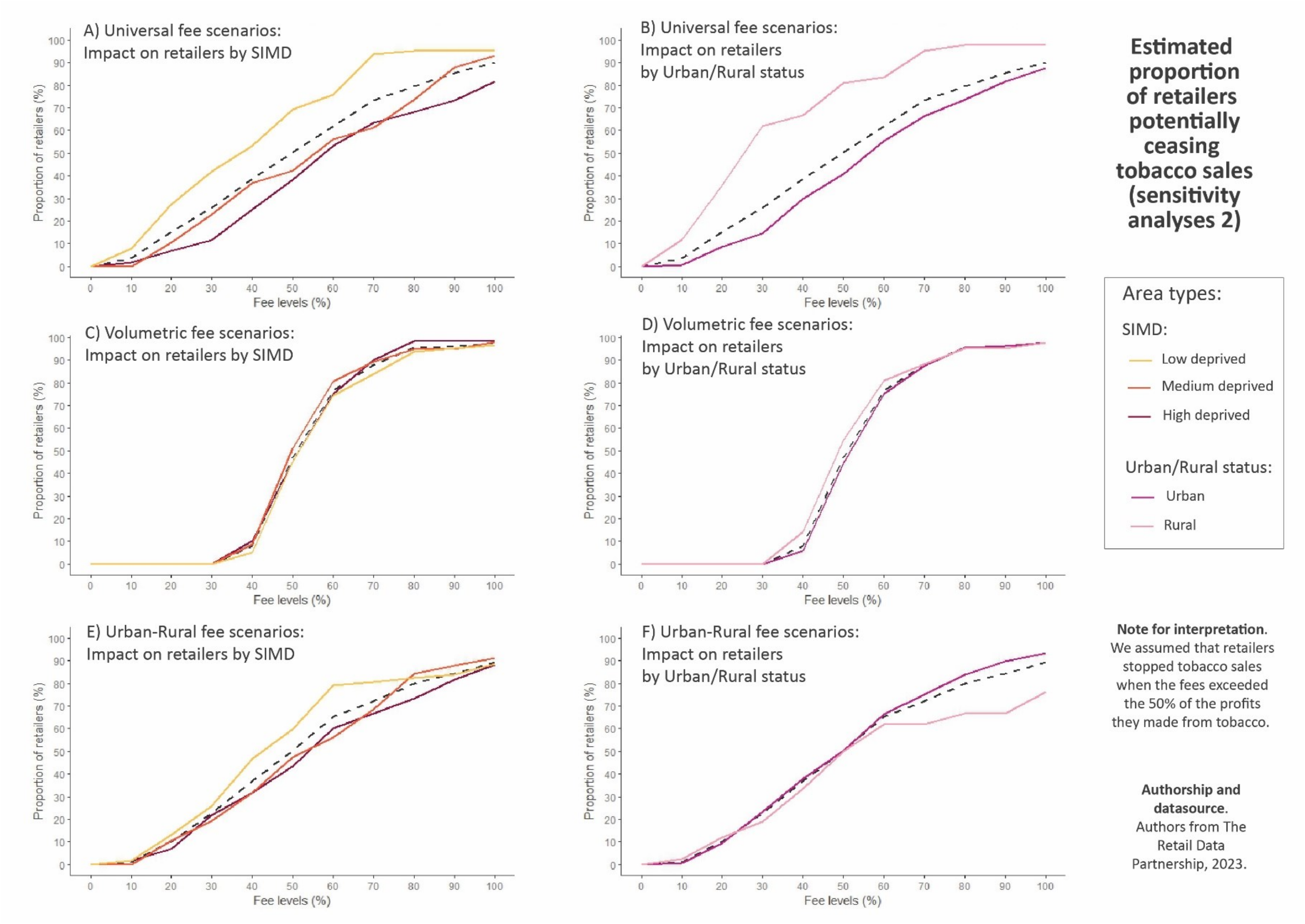

Supplement: Supplementary file 1 [file NIHMS2084466-supplement-Online_Supplement.pdf]
